# Evaluation of a new cemented highly cross-linked all-polyethylene cup: a prospective and randomised study assessing wear and fixation characteristics using radiostereometric analysis

**DOI:** 10.1177/1120700021989991

**Published:** 2021-02-10

**Authors:** Volker Otten, Daniel Wästerlund, John Lindbjörn, Carl Mertens, Sebastian Mukka, Sead Crnalic, Kjell G Nilsson

**Affiliations:** Department of Surgical and Perioperative Sciences, Orthopaedics, Umeå University, Umeå, Sweden

**Keywords:** Highly cross-linked polyethylene, wear, migration, RCT, RSA

## Abstract

**Background and purpose:**

The aim of this prospective, randomised and controlled study was to evaluate the wear and fixation properties of a new cemented highly cross-linked all-polyethylene (HXLPE) cup in comparison with a conventional cemented ultra-high molecular weight polyethylene (ConvPE) cup using radiostereometric analysis (RSA).

**Patients and methods:**

A total of 58 patients (58 hips) with primary osteoarthritis (OA) were enrolled in a randomised controlled trial to receive either a ConvPE cup (control) or HXLPE cup (intervention) with identical geometry. The subjects were randomised in a 1:1 ratio. The primary endpoint was proximal wear measured as femoral head penetration into the cup, secondary outcomes were 3D-wear and annual proximal wear from 1 to 5 years. Cup fixation was measured as movement of the cup in relation to the acetabular bone with proximal migration being the primary outcome measure, 3D-migration and change in inclination as secondary outcomes. The patients were followed for 5 years with RSA performed postoperatively, at 3, 12, 24, and 60 months.

**Results:**

The HXLPE displayed a lower median proximal femoral head penetration compared to ConvPE, with a median difference at 2 years of –0.07 mm (95% CI, –0.10 to –0.04 mm), and –0.19 mm (95% CI, –0.27 to –0.15 mm) at 5 years. Annual proximal wear between 1 and 5 years was 0.03 mm/year for HXLPE and 0.06 mm/year for ConvPE (mean difference 0.05 mm, [95% CI, 0.03–0.07 mm]). Proximal migration, 3D migration and change in inclination was numerically slightly higher for HXLPE, albeit not statistically significant.

**Conclusions:**

Compared to ConvPE, the HXLPE cup displayed significantly lower polyethylene wear. Cup migration was not statistically significant different.

**ClinicalTrials.gov Identifier:**

NCT04322799.

## Introduction

Aseptic loosening is still today the most common indication for revision of the total hip arthroplasty (THA).^1^ Wear particles from the articulating surface are considered to be the main causes of periprosthetic osteolysis, which subsequently leads to loosening of the prosthetic implant.^2^

In an effort to reduce wear, crosslinking the ultra-high molecular weight polyethylene (PE) through high doses of radiation, creating so-called highly cross-linked polyethylene (HXLPE) was proposed.^3^ HXLPE was introduced in THA around the shift of the millennium as an alternative bearing material to ultra-high molecular weight PE, also known as conventional PE (ConvPE), the standard material used in the acetabular cup since the 1960s. Hip simulator studies on HXLPE showed a significant reduction in wear compared with ConvPE.^3,4^ HXLPE is made by crosslinking ConvPE through high dose gamma- or electron beam irradiation. This will, however, create free radicals predisposing the implant to oxidation if not sufficiently eradicated. This is addressed by thermal processing done either by heating the HXLPE above melting temperature or heating up HXLPE to a temperature below the melting temperature in a process known as annealing. Both methods of thermal processing have some drawbacks. Melting has been shown to alter the mechanical properties of the PE, rendering it less resistant to wear and fatigue.^5^ Annealing, on the other hand, will maintain the mechanical properties but will not eliminate all free radicals,^6^ which might put the cup at risk of oxidation, and which indeed has been seen in some retrieved annealed implants.^7^ Finally, the HXLPE is sterilised using gas plasma or ethylene oxide and packed in vacuum or an inert atmosphere. There are different variants of HXLPE on the market; each fabricated using different resins, production methods, x-linking, radical reduction and sterilisation, resulting in different mechanical properties and wear resistance. Several clinical trials have shown a reduction in wear using HXLPE in comparison with ConvPE without increased risk for inferior fixation.^8–11^ However, most studies on HXLPE have been on liners aimed for uncemented metal-backed cups. Studies on HXLPE cups aimed for cemented fixation are sparse, and differences in mechanical properties of the polyethylene may influence on cup fixation.

A new HXLPE has been released by the company Link (X-LINKed, Link, Germany), aimed for both cemented cups as well as for liners for uncemented cups. In an attempt to evaluate this new HXLPE in relation to ConvPE, we performed a randomised controlled trial using RSA. The study was originally planned for 2 years of follow-up. At 2 years there were only small, albeit statistically significant, differences in wear between the implants. However, the pattern of femoral head penetration up to 2 years indicated a potential for larger differences between the 2 types of PE over time. Therefore, it was decided to extend the study up to 5 years.

The aim of this study was to compare the magnitude of wear and to compare the quality of fixation between HXLPE and ConvPE using RSA.^12^

## Materials and methods

### Study setting

This randomised controlled clinical trial was performed between February 2013 and November 2014 at the orthopaedic department, Lycksele Hospital, Sweden. Lycksele Hospital is a level 3 emergency hospital, providing medical care to a catchment area of approximately 40,000 inhabitants.

### Registration

ClinicalTrials.gov (NCT04322799).

### Participants and eligibility criteria

**Figure 1.**
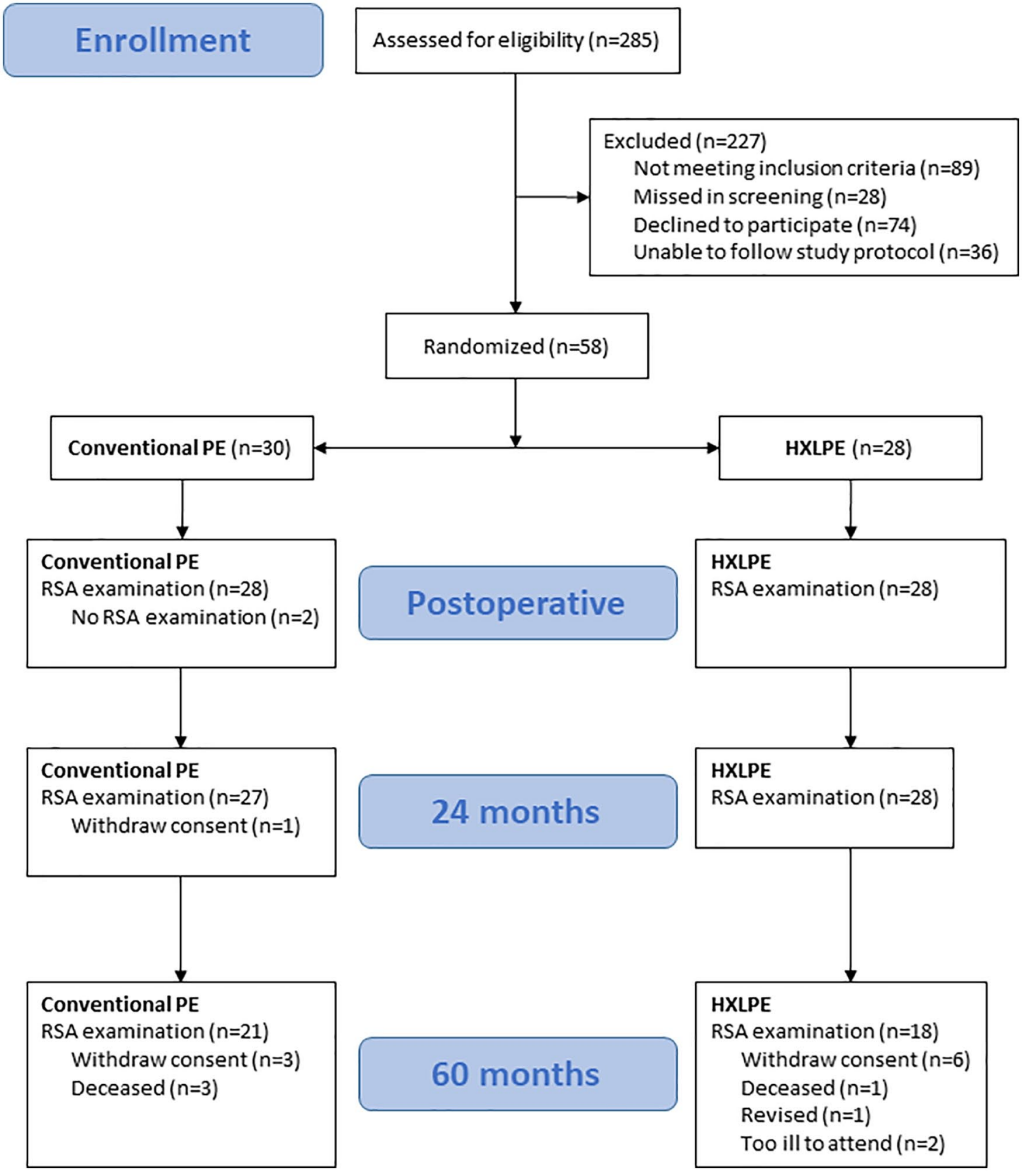
Flowchart according to CONSORT of the study.

We screened all patients deemed suitable for a cemented primary hip arthroplasty for osteoarthritis. All patients were assessed for eligibility according to the inclusion and exclusion criteria (285 patients) (Figure 1). The inclusion criteria were an age between 50 years and older, primary hip osteoarthritis (OA) scheduled for cemented hip arthroplasty, registered in the Västerbotten county and informed consent to participate in the study. Exclusion criteria were rheumatoid arthritis of the affected hip, fracture sequelae, dysplasia, immune suppression or severe systemic illness, significant anatomical abnormalities and malignancy.

Patients were followed-up with RSA measurements performed within the first postoperative week and thereafter at 3, 12, 24 and 60 months.

### Randomisation and blinding

Randomisation was performed in a 1:1 ratio to the control (ConvPE) or intervention (HXLPE) group using concealed envelopes. Randomisation was stratified for gender. Within each stratum, there was block randomisation with 6 permutations in each block. The envelopes were kept separately from the operation theatre and were opened by a research nurse contacted by telephone. The patients, but not hospital staff and research staff were blinded to treatment throughout the study period.

### Implants and surgery

All patients received a cemented Lubinus SP II (LINK, Germany) femoral component with a 32-mm CoCr head. The acetabular component was a cemented Lubinus cup (LINK, Germany), made from either HXLPE (X-LINKed, Link, Germany) or ConvPE. The HXLPE cup is produced by sheet compression and machining GUR 1020 resin. Crosslinking is achieved by a total dose of 75 kGy. The PE sheets are remelted at 150°C after which a layer of at least 5 mm of the surface is shaved off before machining the cup into final shape. The ConvPE cup (LINK, Germany) is also produced from GUR 1020 resin by sheet compression molding and machining but without the irradiation and remelting. The HXLPE cup is sterilised in ethylene oxide whereas the ConvPE cup is gamma sterilised. Both cups are packed in vacuum after sterilisation. All patients were operated in the lateral position using a posterolateral approach and all cups were cemented with Palacos cum Gentamin bone cement (Hereraus Medical, Germany) using 3rd generation cementing technique with 6–8 anchoring holes and cement compression.

The 3 surgeons involved in the study had long experience of using all implants. We used a standard protocol and the patients started rehabilitation on the first postoperative day and were mobilised with full weight-bearing under the supervision of a physiotherapist.

### Radiostereometry and radiological evaluation

Both wear and cup fixation were analysed with RSA. At the time of surgery, a total of 9 tantalum markers (diameter 1 mm) were embedded into the periphery of the cup opening and another 6–9 markers were inserted into the acetabular bone surrounding the cup. RSA measurements were performed within the first postoperative week and thereafter at 3, 12, 24 and 60 months postoperatively. Examinations were performed with the patient in a supine position with simultaneous exposure from 2 x-ray tubes. Analysis of femoral head penetration in relation to the polyethylene cup and cup migration in relation to the acetabulum was calculated using UmRSA software (v6.0, RSA Biomedical, Umeå, Sweden).

#### Polyethylene wear

Femoral head penetration into the polyethylene cup (being a surrogate measure for wear) was measured along three axes; x-axis (medial-lateral), y-axis (proximal-distal), and z-axis (posterior-anterior).

Primary outcome measure for wear was proximal femoral head penetration along the y axis (*“y wear”*).

Secondary measures were; *“xyz wear”* (3D wear or total wear) calculated as the vectorial sum of the femoral head penetration along all three axes, *“xy-wear”* (vectorial sum for x and y axes penetration), and *“yz wear”* (vectorial sum for y and z axes penetration). All measures used the postop RSA examination as reference. Wear rate (mm/year) was calculated as proximal head penetration between 1 and 5 years.

#### Cup fixation

Primary outcome variable for cup fixation was measurement of translation of the centroid of the markers in the polyethylene along the y-axis *(“y migration”* or *“proximal-distal migration”*). Secondary outcome variable was the vectorial sum of centroid translations along all three axes (*“xyz migration”* or *“3D migration”*), and rotations around the z axis (*“z rotation”* or *“increased [+]/decreased [–] inclination”*).

The repeatability of RSA in the current setting was determined on double examinations according to Ranstam et al.,^13^ and the standard deviation (SD) was 0.08 mm for *“y wear”*, 0.14 mm for *“y migration”*, and 0.21° for *“z rotation”*.

### Statistical analysis

To detect a difference ⩾0.18 mm (SD 0.2 mm) in wear between groups (*p* < 0.05) with a power of >80% a total number of at least 36 hips were needed according to pre-study power analysis. To account for potential dropouts during the 5-year follow-up considering the accepted age variation in the inclusion criteria, a total of 58 patients were recruited.

The statistical analyses were based on the intention-to-treat principle. In case of dropouts, the method of last observation carried forward was used.^14^

Data were tested for normal distribution using the Shapiro-Wilks test. In case of normal distribution, the means and standard deviations were calculated and the *t*-test for independent samples were used to compare the different groups. When distribution was non-normal, medians with inter quartile ranges were calculated as well as median differences with 95% confidence intervals (CIs),^15^ and tested with Mann-Whitney U-test. Proportions were analysed with Fisher’s exact test or chi-square test. *p*-values < 0.05 were considered statistically significant.

For group comparisons of *“y migration”* and *“z rotation”* where both negative and positive values are possible, absolute values were used, since the main interest was the magnitude of cup migration. (*“xyz migration”* is a vector length, and thus has only positive values).

For visual description of wear and migration results, graphs displaying median values and interquartile (IQ) ranges were created.

### Ethics

All patients gave informed consent prior to inclusion in the study and the Ethical Review Board Umeå, approved the study (Approval for postop to 2 years: dnr 2011-173-31M, approval for extension 2–5 years: dnr 2018-34-31).

## Results

### Patients

We enrolled 58 patients, 30 in the control group (ConvPE) and 28 in the intervention group (HXLPE). Baseline characteristics of the two groups were similar (Table 1). The distribution of operations between the 3 surgeons did not differ (Table 1). 2 patients in the conventional PE group did not have a postoperative RSA investigation which precluded further RSA investigations. They were, however, followed clinically during the follow-up.

**Table 1.** Patient demographics and distribution of cup sizes.

	Conventional PE (*n* = 30)	HXLPE (*n* = 28)	
Mean age, yrs. (SD)	69 (8)	69 (6)	
Sex, female:male	15:15	14:14	
Operated side, right:left	23:7	12:16	*p* = 0.52^a^
Surgeon 1	8	8	
Surgeon 2	10	14	*p* = 0.26^b^
Surgeon 3	12	6	
Mean height, cm (SD)	172 (9)	171 (10)	
Mean BMI, kg/m^2^ (SD)	29 (4)	28 (5)	
Cup size (mm)			
48	14	12	
50	3	4	
52	6	5	
54	6	5	
56	1	1	
58	0	1	

BMI, body mass index; SD, standard deviation; PE, polyethylene; HXLPE, highly cross-linked polyethylene.

a Fisher’s exact test.

b chi-square test.

56 patients, thus, entered the RSA study. In total, there were 17 dropouts, 16 of which occurred between 2 and 5 years (Figure 1). In the HXLPE group 10 patients were lost between 2 and 5 years. 6 patients withdraw their consent, mainly because high age made travel to the examination site inconvenient. 2 patients were too ill to participate (hepatic cancer and Alzheimer’s disease, respectively) and 1 patient died of causes unrelated to the implant. Finally, 1 patient was revised 3.5 years postoperatively due to loosening of the stem. During revision surgery, the stem could be pulled out by hand while the cup was firmly in place but judged to be fixed by fibrous tissue. This cup had at 2 years a y migration (0.41 mm) and 3D migration (0.89 mm), both values about twice the median of the HXLPE group (0.19 and 0.43 mm, respectively) at 2 years.

In the ConvPE group, 4 patients withdraw their consent, one before the 2-year follow-up, and 3 before the 5-year follow-up, and 3 patients died of causes unrelated to the implant.

### Polyethylene wear

#### Primary endpoint

**Figure 2.**
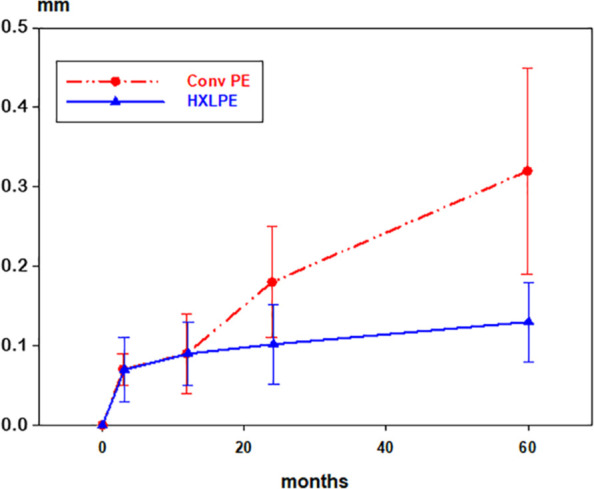
Proximal penetration of the femoral head (*“y wear”*), median and inter quartile range. Red (dashed) line; conventional polyethylene (Conv PE), blue (solid) line; highly cross-linked polyethylene (HXLPE).

The ConvPE group displayed a continuously increasing proximal femoral head penetration (*“y wear”*) during the follow-up period reaching a median of 0.32 (IQ 0.19–0.47) mm at 5 years. In the HXLPE group, on the other hand, the proximal femoral head migration stabilised from 3 months reaching a median of 0.13 (IQ 0.07–0.17) mm at 5 years (Table 2) (Figure 2). Median difference between the groups at 5 years was –0.19 (95% CI, –0.27 to –0.15) mm, (*p* < 0.001).

**Table 2.** Femoral head penetration with postoperative measurement as baseline reference.

		Conventional PE	HXLPE	Median difference (95% confidence interval)	*p*-value^a^
		Median (inter quartile range)			
y wear (mm)^a^	3 m	0.07 (0.03 to 0.08)	0.07 (0.03 to 0.10)	0.01 (–0.01 to 0.02)	0.934
Proximal	12 m	0.09 (0.06 to 0.16)	0.09 (0.04 to 0.13)	−0.01 (–0.03 to 0.01)	0.437
	24 m	0.18 (0.12 to 0.27)	0.12 (0.07 to 0.17)	−0.07 (–0.10 to –0.04)	0.013
	60 m	0.32 (0.19 to 0.47)	0.13 (0.07 to 0.17)	−0.19 (–0.27 to –0.15)	<0.001
xyz wear (mm)^a^	3 m	0.13 (0.09 to 0.17)	0.14 (0.09 to 0.18)	0.02 (–0.02 to 0.08)	0.563
3D–wear	12 m	0.17 (0.10 to 0.23)	0.14 (0.11 to 0.22)	−0.01 (–0.04 to 0.02)	0.647
	24 m	0.23 (0.17 to 0.33)	0.19 (0.13 to 0.24)	−0.04 (–0.09 to –0.01)	0.074
	60 m	0.40 (0.24 to 0.49)	0.20 (0.13 to 0.24)	−0.24 (–0.24 to –0.11)	<0.001
xy wear (mm)^a^	3 m	0.08 (0.06 to 0.11)	0.09 (0.05 to 0.16)	0.02 (–0.02 to 0.03)	0.606
Medial + proximal	12 m	0.11 (0.07 to 0.19)	0.11 (0.07 to 0.15)	−0.01 (–0.03 to 0.03)	0.708
	24 m	0.19 (0.13 to 0.29)	0.15 (0.09 to 0.22)	−0.05 (–0.10 to –0.02)	0.044
	60 m	0.37 (0.20 to 0.48)	0.16 (0.09 to 0.22)	−0.19 (–0.25 to –0.13)	<0.001
yz wear (mm)^a^	3 m	0.11 (0.07 to 0.15)	0.11 (0.07 to 0.16)	0.01 (–0.02 to 0.04)	0.631
Proximal + posterior	12 m	0.11 (0.07 to 0.19)	0.13 (0.10 to 0.19)	−0.01 (–0.04 to 0.05)	0.763
	24 m	0.19 (0.13 to 0.29)	0.17 (0.11 to 0.21)	−0.05 (–0.09 to –0.02)	0.037
	60 m	0.37 (0.20 to 0.48)	0.18 (0.10 to 0.21)	−0.19 (–0.24 to –0.13)	<0.001

PE, polyethylene; HXLPE, highly cross-linked polyethylene.

a Mann-Whitney U-test.

#### Secondary endpoints

Similar patterns and differences in favour of HXLPE were found for the other wear measurements (Table 2). Wear rate from 1 year was for HXLPE a mean 0.01 (SD 0.03) mm/year and for ConvPE mean 0.06 (SD 0.04) mm/year, mean difference 0.05 mm/year (95% CI, 0.03–0.07), *p* < 0.001.

### Cup fixation

**Figure 3.**
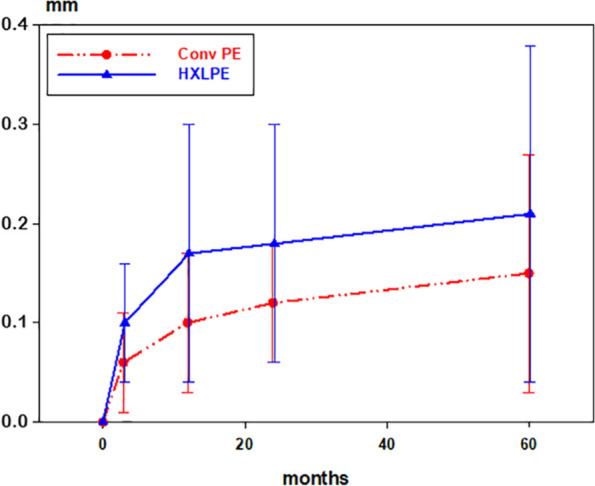
Proximal migration (*“y migration”*) of the cups in the different groups. Red (dashed) line; conventional polyethylene (Conv PE), blue (solid) line; highly cross-linked polyethylene (HXLPE).

Median migration of the cup (*“y migration”*) at 5 years was 0.21 mm for HXLPE and 0.14 mm for ConvPE (*p* = 0.32) (Table 3). The slightly larger migration for HXLPE occurred up to 1 year, thereafter the pattern of migration was similar between the groups (Figure 3). At 2 years 10 HXLPE and 5 ConvPE cups had migrated (“*y migration”*) more than 0.2 mm (*p* = 0.11, Fisher’s test). Also *“3D migration”* and *“z rotation”* was slightly larger for HXLPE, albeit not statistically significant. The proportion of cups displaying increasing or decreasing inclination (*“z rotation”*) did not differ between the groups.

**Table 3.** Cup translations with postoperative measurement as baseline reference.

		Conventional PE	HXLPE	Median difference	*p*-value^a^
		Median (inter quartile range)		(95% confidence interval)	
y migration (mm)	3 m	0.06 (0.04 to 0.14)	0.09 (0.05 to 0.17)	0.02 (–0.01 to 0.04)	0.443
Proximal-distal Absolute values	12 m	0.10 (0.03 to 0.18)	0.17 (0.06 to 0.32)	0.05 (–0.01 to 0.11)	0.219
	24 m	0.12 (0.06 to 0.21)	0.19 (0.07 to 0.31)	0.06 (–0.01 to 0.10)	0.150
	60 m	0.14 (0.07 to 0.30)	0.21 (0.07 to 0.31)	0.05 (–0.02 to 0.09)	0.323
xyz migration (mm)	3 m	0.25 (0.18 to 0.38)	0.20 (0.16 to 0.37)	−0.02 (–0.07 to 0.04)	0.505
3D-migration	12 m	0.31 (0.19 to 0.49)	0.40 (0.22 to 0.77)	0.09 (0.00 to 0.18)	0.213
	24 m	0.31 (0.18 to 0.55)	0.43 (0.24 to 0.78)	0.09 (–0.01 to 0.21)	0.151
	60 m	0.43 (0.28 to 0.69)	0.64 (0.27 to 0.89)	0.13 (–0.02 to 0.29)	0.171
z rotation (degrees)	3 m	0.26 (0.06 to 0.64)	0.25 (0.16 to 0.45)	−0.02 (–0.09 to 0.11)	0.866
abduction/adduction	12 m	0.29 (0.17 to 0.92)	0.42 (0.28 to 0.78)	0.07 (–0.06 to 0.16)	0.552
Absolute values	24 m	0.30 (0.13 to 0.51)	0.54 (0.30 to 0.94)	0.20 (0.08 to 0.42)	0.053
	60 m	0.39 (0.22 to 0.90)	0.64 (0.41 to 1.05)	0.19 (0.03 to 0.38)	0.113

PE, polyethylene; HXLPE, highly cross-linked polyethylene.

a Mann-Whitney U-test.

## Discussion

In this prospective, randomised controlled trial we compared the wear and fixation characteristics of a new cemented HXLPE cup with a cemented ConvPE cup used for many years and with a good clinical track record. In regard to wear, the HXLPE displayed a lower median proximal femoral head penetration compared to the ConvPE with a statistically significant median difference at 2 years of –0.07 mm, increasing to –0.19 mm at 5 years. Also, the mean annual proximal wear between 1 and 5 years was statistically significantly lower for HXLPE. This reduction in wear was not associated with statistically significant inferior fixation of the HXLPE cup.

The step-wise introduction of new implants is gaining grounds in the orthopaedic community to avoid patients’ suffering.^12^ RSA as a method provides an aid in analysing the biological events occurring in the periprosthetic bone. However, RSA data could not supersede a detailed long-term clinical and radiographic follow-up as well and continuing monitoring by national arthroplasty registries.

Previous studies found a correlation between annual wear rate and periprosthetic osteolysis. A wear rate <0.1 mm/year reduces the risk for osteolysis considerably and wear rates below 0.05 mm/year might eliminate osteolysis.^2^ A systematic review comparing different HXLPEs to ConvPE found a pooled mean linear annual penetration rate for HXLPE of 0.042 mm/year (5 brands, 1502 hips.^16^ Using these numbers as reference, the HXLPE in the present study performs better than most HXLPEs available for commercial use in terms of wear (0.01 mm/year). These HXLPEs, however, were intended as liners for use with uncemented, metal-backed cup designs. The data on cemented all-poly HXLPEs is scarce. To our knowledge, only one clinical trial assessing this particular design exists to date comparing a cemented, vitamin-E stabilised highly cross-linked, acetabular cup (VEPE) to ConvPE.^17^

The femoral head penetration in the present study was rather similar for HXLPE and ConvPE up to 1 year, thereafter the penetration stabilised for HXLPE but continued for ConvPE. This resulted in a lower annual wear rate between 1 and 5 years for HXLPE, 0.03 versus 0.06 mm/year. This small but statistically significant difference is reflected in the data from the Swedish Hip Arthroplasty register where the HXLPE cup has a higher survival rate compared to ConvPE.^18^

It is of particular interest to compare the results of the HXLPE in the present study to those of the only other published study with a cemented, all-poly, highly cross-linked acetabular component.^17^ For 3D wear (the only wear parameter documented in the VEPE-trial) a median of 0.20 (IQR 0.13–0.24) mm at 2 years in the present study compares favourably with a mean 0.23 (SD 0.12) mm for the VEPE cup. Thus, for all-polyethylene cups intended for cemented use, crosslinking does indeed reduce the wear rate.

When improving an implant, in this case decreasing the wear rate, there is always a risk of introducing other, untoward, effects. This seems to be the case of the of the highly cross-linked, vitamin E-stabilised all-poly HXLPE cup (VEPE) in the study of Sköldenberg et al.^17^ The authors found a statistically significant higher proximal migration (mean 0.32 mm) of the VEPE cup compared to ConvPE, a higher proportion of VEPE cups displaying proximal migration >0.20 mm, and also significant differences in z-rotation and 3D-migration for the VEPE cup compared to ConvPE. Proximal migration has been proposed as an early warning sign of late aseptic loosening in a systematic review.^19^ The authors put forth a threshold of ⩽ 0.2 mm at 2 years as “acceptable” and classified implants between 0.2–1.0 mm at 2 years as “at-risk”. In the present study the migration along the y-axis (proximal-distal) for the HXLPE cups was a median 0.19 mm, which is below the limit cited above. However, somewhat more cups in the HXLPE group had a proximal migration >0.20 mm. Also, there was a tendency, albeit not statistically significant, for slightly larger 3D-migration and z rotation for the HXLPE cups.

The mechanism behind the numerically slightly larger migration for HXLPE compared to ConvPE is not clear, the difference between the 2 polyethylenes in the present study is the crosslinking, the remelting, and the EtO sterilisation of the HXLPE, all factors which might change some of the PE’s mechanical properties. HXLPE has been found to have a lower elastic modulus and yield strength.^20^ Theoretically, this might affect the quality of the interface between the cup and the acetabulum with forces from the femoral head concentrated through the HXLPE proximally. A reason for the more outspoken migration seen in the VEPE cup of Sköldenberg et al.^17^ might be found in the post-irradiation treatment, the HXLPE of the present study is melted (a 1st generation HXLPE) while VEPE is of the 2nd generation and treated with E-vitamin and annealed as a means to eliminate oxygen radicals. Furthermore, VEPE is *γ*-sterilised unlike the HXLPE in our study which is sterilised using ethylene-oxide.^21^ The amount of radiation used in cross-linking is about the same for the HXLPE used in the current study and the VEPE used in the study of Sköldenberg et al.,^17^ however, another grade of PE was used to make VEPE, (i.e. GUR 1050 vs. 1020 for HXLPE). According to the literature, there are no significant differences in wear and mechanical properties between GUR 1020 and GUR 1050.^22^ How the processes and materials mentioned above might interplay to explain the differences seen in migration between HXLPE, VEPE and ConvPE, is a subject beyond the present study.

While the magnitude of wear is well-studied as a predictor for late revision, the same cannot be said about proximal migration – ideally, there would be a specific threshold rather than an interval. Reasonably, there should be some difference between an implant in the lower scale of the “at-risk” interval described by Pijls et al.^19^ compared to one in the higher scale. Hypothetically, the wear qualities seen in the present HXLPE might make up for the somewhat larger migration seen in our data. However, the uncertainty regarding a specific threshold for proximal migration and risk for later failure, calls for an extended follow-up to monitor these implants, in essence the same conclusion reached in the VEPE-trial.^17^

The strength of this randomised, controlled trial is the use of RSA with high precision and accuracy which allows for the inclusion of small numbers of patients to reach sufficient power, thereby putting as few patients as possible at potential risk. This study was undertaken at a 3rd level hospital production centre under regular clinical conditions in the most commonly encountered cohort of osteoarthritis patients. Surgery was undertaken by surgeons with long experience of cemented acetabular components using the most commonly used cemented cup in Sweden.

A weakness is the relatively large patient dropout between 2 and 5 years. In order to adhere to the intention-to-treat principle, we used the method of Last Observation Carried Forward to deal with missing values.^14^ The Cochrane Musculoskeletal Group deems this method as acceptable in both ‘platinum’ and ‘gold’ level publications included in their reviews.^23^ There are other imputation techniques like that of “multiple imputations” that will generate numbers and sustain power to deal with patients lost to follow-up, this technique, however, requires normally distributed data, and hence, not applicable in our study.^14^

In conclusion, HXLPE reduced polyethylene wear significantly compared to ConvPE. Cup migration was not statistically significantly different.
